# Post-colonoscopy colorectal cancer (PCCRC) rates vary considerably depending on the method used to calculate them: a retrospective observational population-based study of PCCRC in the English National Health Service

**DOI:** 10.1136/gutjnl-2014-308362

**Published:** 2014-11-21

**Authors:** Eva J A Morris, Matthew D Rutter, Paul J Finan, James D Thomas, Roland Valori

**Affiliations:** 1 Cancer Epidemiology Group, Leeds Institute of Cancer & Pathology, University of Leeds, St James's Institute of Oncology, St James's University Hospital, Leeds, UK; 2 University Hospital of North Tees, Hardwick, Stockton on Tees, UK; 3 Colorectal Site Specific Clinical Reference Group, National Cancer Intelligence Network, London, UK; 4 University of Durham, Durham, UK; 5 John Goligher Colorectal Unit, St James's Institute of Oncology, St James's University Hospital, Leeds, UK; 6 National Cancer Registration Service, Northern and Yorkshire Office, St James's Institute of Oncology, St James's University Hospital, Leeds, UK; 7 Gloucestershire Hospitals NHS Foundation Trust, Gloucester, UK

**Keywords:** COLORECTAL CANCER, COLONOSCOPY

## Abstract

**Objective:**

Post-colonoscopy colorectal cancer (PCCRC) is a key quality indicator of colonoscopy. This study compares methods for defining PCCRC rates, proposes a new method of calculating them and quantifies them across the English National Health Service (NHS).

**Design:**

This retrospective observational population-based study involved all individuals with a first primary diagnosis of colorectal cancer made between 2001 and 2010 and treated in the English NHS. Previously published methods for deriving PCCRC rates were applied to the linked routine health data for this population to investigate the effect on the rate. A new method, based on the year of the colonoscopy rather than colorectal cancer diagnosis, was then used to calculate PCCRC rates.

**Results:**

Of 297 956 individuals diagnosed with colorectal cancer, a total of 94 648 underwent a colonoscopy in the 3 years prior to their diagnosis. The application of the published methods and exclusion criteria to the dataset produced significantly different PCCRC rates from 2.5% to 7.7%. The new method demonstrates that PCCRC rates within 3 years of colonoscopy (without exclusions) decreased in the English NHS over 8 years, falling from 10.6% to 7.3% for colonoscopies performed in 2001 and 2007 respectively.

**Conclusions:**

The method used to determine PCCRC rates significantly affects findings with potential to substantially underestimate rates. To enable international benchmarking there needs to be a standardised method for defining PCCRC. This study proposes a new methodology using colonoscopy as a denominator and between 2001 and 2007 this method indicated an 8.6% PCCRC rate across the English NHS. It also demonstrated PCCRC rates have fallen over time.

**Significance of this study**

**What is already known on the topic?**

- Post-colonoscopy colorectal cancer (PCCRC) rates have been proposed as a key quality indicator of a colonoscopy service.
- Several methods of calculating PCCRC rates have been published, with reported rates varying between 2.1% and 7.5%.
- Little is known about PCCRC rates in the English National Health Service (NHS).

**What are the new findings?**

- Rates of PCCRC vary considerably in relation to the method used to define a PCCRC.
- The preferred methodology demonstrated a PCCRC rate within 3 years of colonoscopy of 8.6% in the English NHS.
- PCCRC rates have fallen over time.

**How might it impact on clinical practice in the foreseeable future?**

- The application of a PCCRC indicator for colonoscopy providers based on routine linked NHS data offers a practical method for assessing the quality of the services delivered.
- For international benchmarking an agreed method for defining PCCRC rate is required.

## Introduction

Colorectal cancer is a major public health problem in the UK, with over 40 000 new cases being diagnosed and around 16 000 people dying from the disease every year.^1^ The best survival rates for the illness are observed in those who are diagnosed with early stage tumours^2^ and, as overall survival for the disease is relatively poor in the UK,^3^ ^4^ there is a push to identify more cancers as soon as possible.^5^ Optimising diagnostic services is, therefore, a priority.

The main diagnostic test used to identify colorectal cancer is colonoscopy. Unfortunately, however, the test is not always 100% accurate. Some individuals may undergo colonoscopy which is negative for cancer but subsequently be diagnosed with a cancer—a post-colonoscopy colorectal cancer (PCCRC). This may be a result of the development of a rapidly growing new tumour that was not present at initial colonoscopy but, more frequently, it appears to be due to missed or inadequately excised pre-cancerous lesions, or the colorectal cancer simply being missed by the colonoscopist.^6^ ^7^ The rate of PCCRC occurrence has been proposed, therefore, as a quality indicator of a colonoscopy service.

Generating a robust indicator of PCCRC rates is challenging. Several studies have sought to quantify the occurrence of PCCRCs and determine what factors predispose to their development.^7–17^ Across these studies PCCRCs were consistently seen to be more common in older age groups, in women, in the proximal bowel and following colonoscopies undertaken by non-specialist endoscopists.^10^ ^16^ ^18^ ^19^ The reported rates of PCCRC have varied considerably from 2.1% to 7.5% and although this could be due to differences in the quality of colonoscopic services across the populations considered, it may also be a reflection of the different datasets and methods used to calculate the rates.

The National Cancer Data Repository (NCDR)^20^ contains information on all individuals diagnosed with colorectal cancer in England and their hospital management (including the colonoscopic investigations). Therefore, it enables population and organization based investigation of patterns of PCCRCs across the English National Health Service (NHS). This study aimed to make use of these data to investigate how PCCRC rates vary in relation to the method adopted to derive them, to determine the optimal methodology to calculate PCCRC rates and, using this technique, to investigate any variability and factors associated with the occurrence of PCCRC across the English NHS.

## Methods

All individuals with a first diagnosis of colorectal cancer (International Classification of Diseases V.10 (ICD10)^22^ code C18–20) between 2001 and 2010 were identified in the NCDR. The hospital records of these individuals were then sought to identify all colonoscopies (codes of eligible procedures listed in table 1) undertaken prior to their diagnosis. Two datasets were then extracted. The first used cancers as the denominator and contained data on the first primary diagnosis of colorectal cancer in an individual made over the study period. The second used colonoscopies as the denominator and included information on each of the colonoscopies undertaken in individuals who went on to have a diagnosis of colorectal cancer within 3 years of this test.

**Table 1:** OPCS4 codes used to identify a colonoscopy

OPCS4 code	Description
G79	Therapeutic operations on ileum
G80	Diagnostic endoscopic examination of ileum
H20	Endoscopic extirpation of lesion of colon
H21	Other therapeutic endoscopic operations on colon
H22	Diagnostic endoscopic examination of colon

The patient-level data in both datasets included age at diagnosis and colonoscopy, sex, socioeconomic status (based on quintiles of the income domain of the Index of Multiple Deprivation (IMD) 2007) and Charlson comorbidity score^21^ (based on diagnostic reasons for hospital admissions in the year prior to diagnosis of the cancer). In addition, all individuals who had experienced a hospital admission for Crohn's disease, ulcerative colitis or diverticular disease were identified (ICD10 codes K50, K51 and K57 respectively). Tumour information included date of diagnosis, modified Dukes’ stage of disease at diagnosis and site of tumour based on ICD10 codes. These were classified as rectum or sigmoid colon (ICD10 C187, C19–20), splenic flexure and descending colon (C185–6), transverse colon (C184), right colon (C180–3) or colon not otherwise specified (C188–9).

In both datasets the interval between each colonoscopy and the diagnosis of the subsequent cancer was determined and grouped into two categories. In the cancer dataset, tumours that were diagnosed within 6 months of a colonoscopy were classed as ‘detected’ cancers, while those diagnosed more than 6 months after the colonoscopy were classed as PCCRC. All other cancers (in individuals with no history of colonoscopy at the time of cancer diagnosis or in the 36 months prior to diagnosis) were allocated to a ‘no colonoscopy’ group. Likewise, in the colonoscopy dataset, colonoscopies following which a cancer was diagnosed within 6 months were identified as true positive colonoscopies and those in which a cancer was diagnosed greater than 6 months after the investigation were deemed false negative colonoscopies. It is appreciated that the terms false negative and true positive normally depend on there being a gold standard which does not exist for the diagnosis of colorectal cancer and its precursors. In this study it is presumed that colonoscopy has the potential to prevent or detect all cancers that might otherwise present within 3 years; thus the appearance of cancer within 3 years is the gold standard.

Some individuals underwent multiple colonoscopies in the detected and PCCRC periods. This was flagged in the cancer dataset, and only the closest colonoscopy to diagnosis in each category was used in analyses. However, individual records for each colonoscopy remained in the colonoscopy dataset and the total number of colonoscopies an individual underwent in either the true-positive or false-negative category was noted.

**Figure 1:**
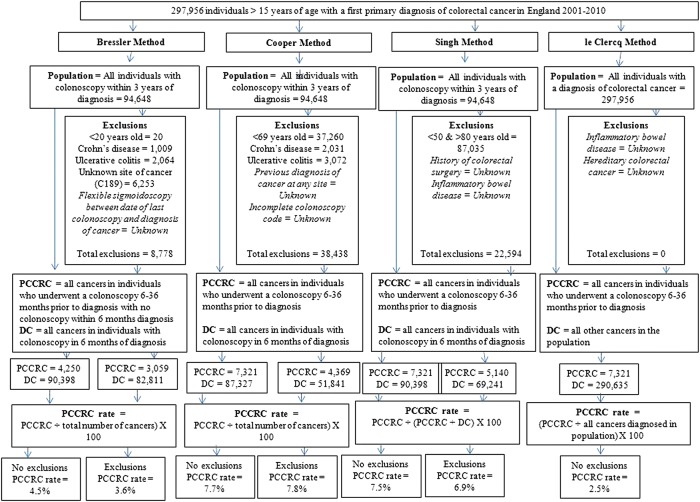
Application and results of four previously published methods for determining post-colonoscopy colorectal cancer (PCCRC). Note: the original le Clercq study calculated PCCRCs over a 0–60-month time period whereas all the other studies used the period of 0–36 months. In the interests of consistency and to enable more ready comparison of methods, in this particular study, the le Clercq method has been simplified to calculate PCCRCs covering a period of 0–36 months too.

Four previously published population-based methods for deriving PCCRC rates had used the total number of individuals with cancers as the denominator.^7^ ^10^ ^11^ ^16^ The methods adopted were discussed with the authors of three papers before application to this cancer dataset.^7^ ^10^ ^16^ These different methods had all made exclusions from their study populations (as detailed in figure 1) and so, if possible, individuals in our study population who would have been excluded following these methods were identified and flagged. Due to the data available in the NCDR it was not possible, however, to apply all the criteria for all the methods. PCCRC rates were then derived based on the four different methods described below and in figure 1.

### Bressler method^10^

This method defined PCCRCs as those that occurred in individuals who had a colonoscopy 6–36 months prior to diagnosis of their colorectal cancer but who did not have a colonoscopy within 6 months of their diagnosis. This approach effectively precluded any patients diagnosed with colonoscopy being designated as PCCRC. Thus only cancers diagnosed with other methods could be labelled as PCCRC. The PCCRC rate was defined as the number of PCCRCs divided by the total number of individuals with a cancer who underwent any colonoscopy prior to diagnosis.

### Cooper method^11^

This method defined PCCRCs as all those that occurred 6–36 months prior to diagnosis of colorectal cancer in individuals irrespective of whether their tumour was subsequently identified by a colonoscopy. Again, the PCCRC rate was defined as the number of PCCRCs divided by the total number of individuals with a cancer who underwent any colonoscopy in the 36 months prior to diagnosis.

### Singh method^16^

Like Cooper *et al*, this method defined PCCRCs as all those that occurred 6–36 months prior to diagnosis of colorectal cancer in individuals irrespective of whether their tumour was subsequently identified by colonoscopy. But the method also allowed cancers to be counted in both the PCCRC and detected categories so if an individual underwent two colonoscopies, one in the 6 months prior to diagnosis and another in the 6–36 month period, a PCCRC and a detected cancer were included in the rate calculation. The denominator was all PCCRCs plus all detected cancers.

### Le Clercq method^7^

This method defined a PCCRC in the same way as Cooper and Singh but used all individuals with cancer, rather than just those who had a colonoscopy prior to, or at, diagnosis, as the denominator.

### New colonoscopy method

The colonoscopy dataset was then analysed using a new method which changed the denominator from cancers to colonoscopies. The purpose of creating this new method was to have a measure that was most relevant to patients having a colonoscopy (not to those who have a cancer) by creating a measure based on the number of colonoscopies, not cancers. It is presumed that patients who have a cancer currently, or are destined to develop one in the next 3 years, would like to know the likelihood the cancer is missed or not prevented by the current colonoscopy. On the basis of this presumption the proposed PCCRC rate uses the appearance of cancer over 3 years as the gold standard: the true positives plus the false negatives. The PCCRC rate is defined simply as the number of false-negative colonoscopies divided by the gold standard. For individuals who underwent multiple colonoscopies only the first true-positive and first false-negative colonoscopy was included in this calculation.

Trends in PCCRC rates in relation to the year of colonoscopy were investigated. The follow-up time in which a cancer could be diagnosed for colonoscopies undertaken at the beginning of the cohort was greater than for those undertaken at the end. Prior to investigating the characteristics of PCCRC and detected cancers using this method the data were, therefore, censored to include only those that occurred within 3 years of the initial colonoscopy.

Multilevel (random effects) logistic regression models were built to determine factors associated with the occurrence of a PCCRC. These models were built with a hierarchy of colonoscopies clustered within individuals. The dependent variable was the occurrence of a PCCRC and the explanatory variables were year of colonoscopy, age at colonoscopy (per year increase), sex of individual, site of tumour, IMD^23^ income category at diagnosis, and prior in-patient hospital admission for Crohn's disease, ulcerative colitis and diverticular disease.

Finally, the statistical analysis was restricted to cancers appearing within 36 months of a colonoscopy. The data (for colonoscopies performed up to the end of 2005 only) were further analysed to 60 months to determine whether new PCCRCs continue to form 2–5 years after a colonoscopy.

## Results

Between 1 January 2001 and 31 December 2010, 297 956 individuals in England were identified within the NCDR as being diagnosed with a first primary colorectal cancer. Within this population, 94 648 (31.7%) individuals had a record of a colonoscopy within the 3 years prior to their diagnosis. Of these cancers, 90 398 had one or more colonoscopies in the 6 months prior to diagnosis and 7321 had a colonoscopy within the 36 to 6 months preceding diagnosis. This population forms the basis of the cancer dataset used to compare the previously published methods, and its characteristics are described in table 2.

**Table 2:** Characteristics of the cancers diagnosed between 2001 and 2010 in relation to colonoscopy category

	Cancers occurring								
	>6 months after colonoscopy		≤6 months after colonoscopy		With any colonoscopy		Without colonoscopy		
Characteristic	n	%	n	%	n	%	n	%	All cancers
Median age at diagnosis (IQR)	73 (65–80)		72 (64–79)		73 (64–81)		72 (64–79)		73 (64–80)
Sex									
Male	3900	2.4	51 545	31.6	53 680	32.9	109 649	67.1	163 329
Female	3421	2.5	38 853	28.9	40 968	30.4	93 659	69.6	134 627
IMD income category at diagnosis									
Most affluent	1532	2.5	18 954	31.1	19 815	32.5	41 085	67.5	60 900
2	1553	2.4	19 656	30.1	20 543	31.5	44 704	68.5	65 247
3	1473	2.3	19 241	30.3	20 113	31.6	43 437	68.4	63 550
4	1484	2.5	17 682	30.2	18 540	31.7	39 979	68.3	58 519
Most deprived	1279	2.6	14 865	29.9	15 637	31.4	34 103	68.6	49 740
Charlson comorbidity score at diagnosis									
0	4497	2.2	62 794	30.2	65 363	31.4	142 892	68.6	208 255
1	1284	3.1	13 681	32.7	14 444	34.5	27 457	65.5	41 901
2	926	3.0	9459	30.9	9989	32.7	20 576	67.3	30 565
≥3	614	4.3	4464	31.4	4852	34.1	9362	65.9	14 214
Unknown	0	0.0	0	0.0	0	0.0	3021	100.0	3021
Previous hospital admission for Crohn's disease									
No	6987	2.4	89 561	30.3	93 639	31.6	202 286	68.4	295 925
Yes	334	16.4	837	41.2	1009	49.7	1022	50.3	2031
Previous hospital admission for ulcerative colitis									
No	6493	2.2	88 736	30.2	92 584	31.5	201 670	68.5	294 254
Yes	828	22.4	1662	44.9	2064	55.8	1638	44.2	3702
Previous hospital admission for diverticular disease									
No	4380	1.9	63 230	26.8	65 822	27.9	170 200	72.1	236 022
Yes	2941	4.7	27 168	43.9	28 826	46.5	33 108	53.5	61 934
Dukes’ stage at diagnosis									
A	1113	3.8	12 865	44.3	13 340	45.9	15 718	54.1	29 058
B	1517	2.1	25 474	35.5	26 205	36.5	45 534	63.5	71 739
C	1496	2.1	23 193	32.3	24 030	33.5	47 797	66.5	71 827
D	995	2.1	10 402	22.4	11 086	23.8	35 428	76.2	46 514
Unknown	7321	9.3	18 464	23.4	19 987	25.4	58 831	74.6	78 818
Tumour site									
Rectal/sigmoid colon	2926	1.7	46 552	27.6	48 372	28.6	120 518	71.4	168 890
Splenic flexure/descending colon	289	2.1	4331	31.2	4452	32.1	9409	67.9	13 861
Transverse colon	398	3.0	5039	38.3	5212	39.6	7945	60.4	13 157
Right colon	2679	3.7	29 001	39.8	30 359	41.7	42 530	58.3	72 889
Colon NOS	1029	3.5	5475	18.8	6253	21.4	22 906	78.6	29 159
Total	7321	2.5	90 398	30.3	94 648	31.8	203 308	68.2	297 956

IMD, Index of Multiple Deprivation; NOS, not otherwise specified.

The process and results of the application of the four previously published methods of determining PCCRC rates is shown in figure 1. Using the Bressler method, but without any exclusions, 4250 of these cancers were classed as PCCRC leading to a 4.5% PCCRC rate. In contrast, both the Cooper and Singh methods increased the number of cancers classed as PCCRC to 7321 but, due to the differences in how the denominator was derived for each method, the PCCRC rates were 7.7% and 7.5% respectively. The le Clercq method also identified 7321 PCCRCs but the denominator included all cancers diagnosed in the study region, irrespective of whether they were diagnosed by colonoscopy or another method. This significantly increased the denominator and so reduced the PCCRC rate to 2.5%. The rates derived by each of these methods following the application of the original exclusion criteria used in the studies are, again, shown in figure 1. Significant variation was observed in rates (2.5–6.8%) depending on the exclusion criteria adopted.

**Figure 2:**
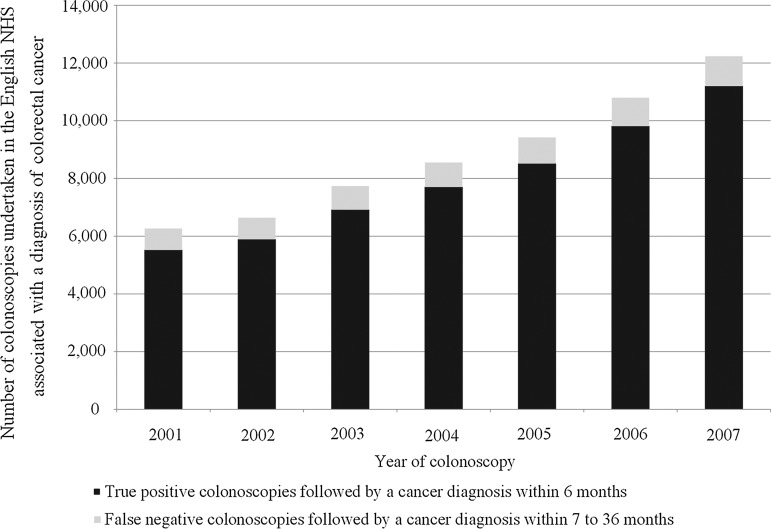
Number of colonoscopies undertaken in the English National Health Service (NHS) followed by a diagnosis of cancer within 36 months.

The data in the colonoscopy dataset had a different structure as it used colonoscopy as the denominator. Therefore, it contained information on the 61 633 colonoscopies undertaken between 2001 and 2007 in the 57 963 individuals who went on to be diagnosed with a colorectal cancer within 3 years of their investigation. Of these colonoscopies 55 539 were true positive and 6094 false negative. When only the colonoscopy closest to diagnosis was included for an individual with multiple investigations in either or both the true-positive and false-negative categories, these numbers reduced to 52 992 and 4971 respectively. The characteristics of this population are shown in table 3. The number of cancers diagnosed by colonoscopy increased significantly over time (figure 2), in keeping with the improved access to colonoscopy and the increase in colonoscopy activity over these years. Cancers arising following false-negative colonoscopies were significantly more common in women, in the right colon and in those with greater comorbidity.

**Table 3:** Characteristics of the individuals and cancers occurring within 3 years of a colonoscopy undertaken between 2001 and 2007

	Cancers occurring in individuals				
	>6 months after colonoscopy		≤ 6 months after colonoscopy		All cancers
Characteristic	n	%	n	%	n
Median age at diagnosis (IQR)	72 (64–69)		74 (65–81)		72 (64–79)
Sex					
Male	2625	8.1	29 755	91.9	32 380
Female	2346	9.2	23 237	90.2	25 583
IMD income category at diagnosis					
Most affluent	1009	8.7	10 548	91.3	11 557
2	1101	8.8	11 454	91.2	12 555
3	986	8.0	11 382	92.0	12 368
4	1012	8.6	10 742	91.4	11 754
Most deprived	863	8.9	8866	91.1	9729
Charlson comorbidity score at diagnosis					
0	3042	7.5	37 431	92.5	40 473
1	873	10.6	7366	89.4	8239
2	648	10.1	5745	89.9	6393
≥3	408	14.3	2450	85.7	2858
Previous in-patient hospital episode for Crohn's disease					
No	4743	8.3	52 496	91.7	57 239
Yes	228	31.5	496	68.5	724
Previous in-patient hospital episode for ulcerative colitis					
No	4392	7.8	51 943	92.2	56 335
Yes	579	35.6	1049	64.4	1628
Previous in-patient hospital episode for diverticular disease					
No	2954	7.2	37 971	92.8	40 925
Yes	2017	11.8	15 021	88.2	17 038
Number of colonoscopic investigations in time period					
1	4223	7.7	50 669	92.3	54 892
>1	748	24.4	2323	75.6	3071
Dukes’ stage at diagnosis					
A	732	9.5	6968	90.5	7700
B	1029	6.3	15 213	93.7	16 242
C	994	7.0	13 178	93.0	14 172
D	686	10.2	6071	89.8	6757
Unknown	1530	11.7	11 562	88.3	13 092
Tumour site					
Rectal/sigmoid colon	1897	6.7	26 349	93.3	28 246
Splenic flexure/descending colon	206	7.4	2580	92.6	2786
Transverse colon	276	8.6	2923	91.4	3199
Right colon	1859	9.8	17 916	94.0	19 055
Colon NOS	733	15.7	3944	84.3	4677
Total	4971	8.6	52 992	91.4	57 963

IMD, Index of Multiple Deprivation; NOS, not otherwise specified.

**Figure 3:**
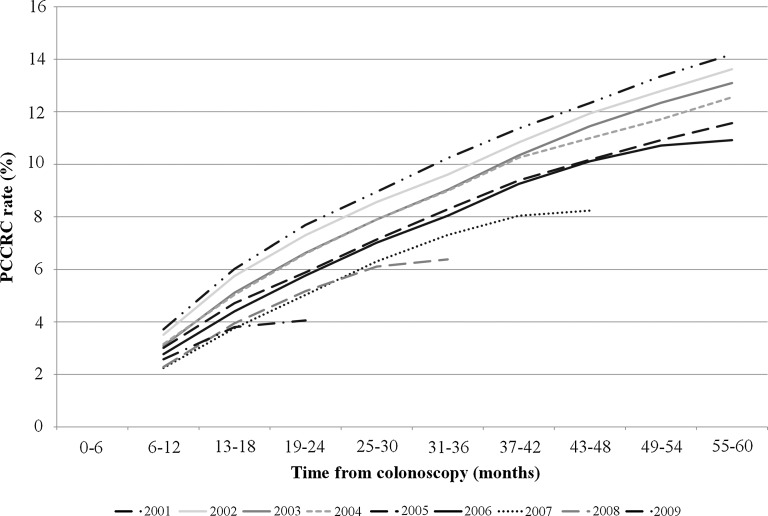
Post-colonoscopy colorectal cancer (PCCRC) rates over time in the English National Health Service (NHS).

The new method to determine PCCRC rates in a number of time intervals after colonoscopy undertaken in each calendar year was then applied. For example, for colonoscopy undertaken in 2001 there were 5281 investigations that led to a detected cancer and so were deemed a ‘true-positive’ colonoscopy. But in the 6–12 months following colonoscopies in this year, 204 cancers were diagnosed, indicating 204 false-negative colonoscopies. The rate of PCCRC at this time point was, therefore, 204 divided by 5485 (the total of true-positive (5281) and false-negative (204) colonoscopies), giving a PCCRC rate of 3.7%. Likewise, with 3 years of follow-up for colonoscopies undertaken in 2001, a total of 602 false-negative tests had occurred. At this time point, therefore, the PCCRC rate was 10.2%. Identical calculations were undertaken for each colonoscopy year at 6 month time intervals and the results of these analyses are shown in figure 3.

Overall, the PCCRC rate was 8.6% at 3 years of follow-up for colonoscopies undertaken between the beginning of 2001 and the end of 2007. Figure 3 illustrates how the PCCRC rate based on the colonoscopy dataset has changed up to 5 years following colonoscopy during the period of the study. The 5-year rate fell from 14.2% in 2001 to 8.2% in 2005. The 3-year rate fell from 10.2% in 2001 to 7.3% in 2007. Finally, the 1-year rate fell from 3.7% in 2001 to 2.6% in 2009.

Table 4 shows the results of a multivariable analysis investigating the odds of developing a PCCRC within 3 years of a colonoscopy. There was a 6% reduction in the odds of development of a PCCRC following colonoscopy for each year the study advanced (OR 0.94, 95% CI 0.93 to 0.95, p<0.01). In contrast, the risk was increased in women compared with men (OR 1.13, 95% CI 1.06 to 1.20, p<0.01) and in those with previous hospital admissions for Crohn's disease (OR 2.54, 95% CI 2.11 to 3.06, p<0.01), ulcerative colitis (OR 5.82, 95% CI 5.17 to 6.54, p<0.01) and diverticular disease (OR 1.86, 95% CI 1.75 to 1.98, p<0.01). There was a marked increase in the odds of developing a PCCRC in individuals who underwent multiple colonoscopies (OR 2.93, 95% CI 2.71 to 3.15, p<0.01).

**Table 4:** Odds of the development of a PCCRC within 3 years of a colonoscopy

Characteristic	OR	Lower 95% CI	Upper 95% CI	p Value
Year of colonoscopy	0.94	0.93	0.95	<0.01
Sex				
Male	1.00			
Female	1.13	1.06	1.20	<0.01
Age (per year)	1.00	0.99	1.00	<0.01
IMD income category				
Most affluent	1.00			
2	1.01	0.92	1.11	0.76
3	0.92	0.84	1.01	0.09
4	0.99	0.90	1.09	0.90
Most deprived	1.01	0.92	1.12	0.79
Previous hospital admission for Crohn's disease				
No	1.00			
Yes	2.54	2.11	3.06	<0.01
Previous hospital admission for ulcerative colitis				
No	1.00			
Yes	5.82	5.17	6.54	<0.01
Previous hospital admission for diverticular disease				
No	1.00			
Yes	1.86	1.75	1.98	<0.01
Site of tumour				
Rectum/sigmoid colon	1.00			
Splenic flexure/descending colon	0.98	0.84	1.15	0.82
Transverse colon	1.17	1.02	1.35	0.02
Right colon	1.42	1.32	1.52	<0.01
Colon NOS	2.34	2.13	2.58	<0.01
Number of colonoscopies in PCCRC/detected category (per colonoscopy)	2.93	2.71	3.15	<0.01

IMD, Index of Multiple Deprivation; PCCRC, post-colonoscopy colorectal cancer; NOS, not otherwise specified.

## Discussion

This is the first population-based study investigating the occurrence of PCCRC across the English NHS. It has shown that the PCCRC rate varies considerably in relation to the method used to define a PCCRC and the exclusions applied to the dataset. The application of four previously published methods led to rates ranging from 2.5% to 7.7% using the same dataset. This means that published data on PCCRC cannot be used to compare the quality of colonoscopy between jurisdictions and rates derived from different methodologies may not be comparable.^24^ Meta-analyses of PCCRC or interval cancers that do not take account of the different methodologies used to calculate rates will be flawed.^24^ To enable comparisons there needs to be agreement on a single methodology or adjustments need to be made, as has been done in this study. After adjusting for differences in methodology, the rates of PCCRC in the English NHS are similar to those in other countries.

All the previously published methods of calculating PCCRC are based on a denominator of the number of cancers diagnosed in a population. To provide patients with a measure focused on the colonoscopy rather than the cancer, a new method is presented using colonoscopies as the denominator. The new method summates detected cancer and PCCRC to provide a gold standard measure for colonoscopy against which PCCRC can be compared. Unlike the gold standard of purely diagnostic tests, this gold standard takes account of the therapeutic capacity of colonoscopy: its ability to prevent, not just detect cancer. This new approach provides the patient who has a cancer, or is destined to present with one in the next 3 years, with a probability the cancer will be missed or not prevented. If rapidly growing cancers with no precursor lesions exist, this rate will never become zero. However, with studies demonstrating that >75% of PCCRCs are either missed or preventable,^25^ and with ever improving quality of colonoscopy, it is expected that future studies will demonstrate the rate of PCCRC (using this method) could be as low as 1%. This proposed figure of 1% is arrived at by first assuming that PCCRC rates will fall from the 2007 rate of 7.3% to at least 4%. If more than 75% of PCCRCs are either missed or preventable, then with an observed PCCRC rate of 4% we might assume 1% to be unavoidable. This lowest achievable PCCRC rate would then represent the ‘inevitable’ rate of cancer following colonoscopy no matter how well the procedure is done. Patients do not find it difficult to grasp the concept of rapidly growing cancers (indeed most are surprised at how slowly colorectal cancers develop). Thus patients are likely to understand that fast growing cancer is, sometimes, inevitable after a negative colonoscopy and find acceptable a sensitivity of 99% of either detecting cancer or preventing it developing in the next 3 years.

An alternative denominator of a patient-centric quality measure to that proposed is all colonoscopies. The resultant statistics would be of the order of 0.05% or 1:2000 (assuming a colonoscopy cancer detection rate of 1% and a PCCRC rate of 5%)—a difficult number for patients to grasp compared with 5% or 1:20 for the PCCRC rate using the proposed gold standard. A further factor to consider is the CI for either measure. Many thousands of procedures or hundreds of cancers are required to provide robust estimates of rates.^26^ Thus reporting in reasonable timespans (eg, annually) can only occur for procedure volumes in endoscopy units or across regions or nations; the sample size will be too small for monitoring performance of individuals when surrogate measures such as adenoma detection rate, a good proxy for interval cancer or PCCRC, are an acceptable alternative.^13^ ^27^

The new proposed approach yielded a PCCRC rate at 3 years post colonoscopy of 8.6% in the first 7 years of the study period. The new method allows prospective assessment of changes in PCCRC rates over time following colonoscopy and the rates relate to the time the procedures were performed, not years afterwards. It has demonstrated that in the English NHS, rates have declined over time with the 3-year rate falling from 10.2% in 2001 to 7.3% in 2007.

This study, as in others, identified several factors associated with the development of a PCCRC. A greater proportion of PCCRCs were found in the right colon.^7^ ^10^ ^11^ ^16^ ^28^ The underlying reasons for this are likely to be multifactorial. For example, for a colonoscopy to be successful it is important for the endoscopist to visualise the entire colon and reach the caecum, but the proximal colon is difficult to reach in some patients and sometimes the landmarks are unclear so colonoscopists may not have reached the caecum even though they think they have. Furthermore, this portion of the colon is more difficult to cleanse with oral agents, making lesions more difficult to identify. Finally, a greater proportion of tumours on the right side of the bowel are associated with microsatellite instability.^29–32^ Such tumours are thought to be fast growing and associated with precursor sessile serrated lesions that can be difficult to detect at colonoscopy.^29^ All these factors may, therefore, lead to a preponderance of right-sided PCCRC.

The increase in the odds of occurrence of a PCCRC in those with inflammatory bowel disease (IBD) and diverticulosis has been found in other studies.^10^ ^11^ ^16^ The increased risk of PCCRC with IBD may be explained by malignancy being more difficult to detect in the context of a diseased colon because of concurrent abnormalities (such as inflammatory polyps) and because cancer in IBD can have a different underlying morphology. Finally, IBD-related cancer may be more aggressive and develop much quicker. Greater technical difficulty performing the procedure may explain the higher risk of PCCRC in patients with diverticular disease. These reasons may also explain the major increase in odds of PCCRC for those undergoing multiple colonoscopies. Those individuals undergoing multiple investigations are likely to be under surveillance for an increased risk of cancer or have experienced an incomplete colonoscopy that needed to be repeated. The strong association of multiple colonoscopies and the development of a PCCRC is again, therefore, not unexpected. These findings, in keeping with other studies, emphasise how vigilant the colonoscopist has to be when colonoscoping these patients. Finally, if PCCRC rates in patients with bowel disease and in those having repeated colonoscopies cannot be improved, patients should be warned of the greater chance of development of PCCRC.

The main limitations of this study centre on the fact that it is based upon routine data. This reduces the resolution of the study. For example, it is not possible to definitively determine whether a cancer is truly a PCCRC or a detected cancer but rather this has to be inferred from the time interval between the colonoscopy and the date of diagnosis of the tumour. Likewise, it is not possible to definitively distinguish between individuals with and without conditions leaving them at a high risk of developing cancer or between colonoscopies undertaken as a part of a surveillance or screening programme and those undertaken for symptomatic reasons. The National Cancer Intelligence Network who developed the NCDR recognise these limitations and are actively seeking to address them by extending the resource to hold an enhanced cancer registry dataset and as many routine health datasets as possible. By including information from primary care, screening and other similar datasets it is intended its scope will be increased to capture more detailed information about the patient, their colonoscopy and the reasons behind it. This will strengthen the new methodology proposed and address these limitations.

This study is based on the data held within the NCDR.^20^ This resource holds population-based data on individuals diagnosed with cancer in England and the routine and administrative nature of these datasets does, unfortunately, pose some limitations. For example, the quality and accuracy of the Hospital Episode Statistics (HES) component of the NCDR (from which the colonoscopy information has been derived) has been questioned, and for colorectal cancer there are around 8% more cancers listed in this dataset than are identified by cancer registries (personal communication NCIN). As the National Cancer Registration Service over this time period has a proven ascertainment rate of >98%^33^ this would suggest that there is significant miscoding in HES (perhaps as a result of the suspicion of cancer rather than confirmed cancer) and relying on this resource alone to identify PCCRC would overestimate the rate. This study has, however, relied on linked data and this linkage has ensured the accuracy of the cancer diagnosis. Furthermore, comparisons of the information held in HES to that collected by clinical trials suggests that agreement is good for cases of confirmed cancer.^35^ As a result, such linked data will provide the most robust figures possible.

Another limitation of the NCDR is that, currently, it only contains information on colonoscopies in individuals who go on to be diagnosed with cancer. As the clinical thresholds for use of colonoscopy and the incidence of colorectal cancer vary across the country, the optimal method of assessing the sensitivity and specificity of colonoscopy would include all colonoscopies rather than just those that led to cancer. Furthermore, high-quality colonoscopy can also reduce the incidence of the disease by the identification and removal of any precancerous lesions. The most robust method to assess PCCRC rates would be to incorporate information on all colonoscopies undertaken in the NHS, rather than just those in individuals who subsequently went on to be diagnosed with cancer.

Although colonoscopy is the main diagnostic investigation for colorectal cancer, flexible sigmoidoscopy is also commonly used in England. This technique allows visualisation of the bowel up to the splenic flexure and its adoption has been shown to reduce the incidence of left-sided lesions and reduce mortality from colorectal cancer.^36^ It is now being assessed within the NHS Bowel Cancer Screening Programme. These analyses do not take into account the use of this technique but rather focus solely on colonoscopy. Further work is required, therefore, to assess the PCCRC rate of flexible sigmoidoscopy.

In conclusion, this study has demonstrated that the method used to determine PCCRC rate significantly affects findings, thus international benchmarking requires an agreed method for calculating PCCRC. The new method proposed uses colonoscopy as the denominator and its application demonstrates PCCRC rates are falling with time in the English NHS.
